# Analysis of the health advocacy concept from the perspective of the evolutionary method

**DOI:** 10.1590/1980-220X-REEUSP-2023-0170en

**Published:** 2023-10-13

**Authors:** Odaleia de Oliveira Farias, Maria Gabriela Miranda Fontenele, Francisca Elisângela Teixeira Lima, Marli Teresinha Gimeniz Galvão, Viviane Martins da Silva, Marcos Venícios de Oliveira Lopes

**Affiliations:** 1 Universidade Federal do Ceará, Departamento de Enfermagem, Fortaleza, CE, Brazil.

**Keywords:** Health Advocacy, Nursing, Concept formation, Health promotion, Evidence-based nursing, Defensa de la Salud, Enfermería, Formacíon de concepto, Promoción de la salud, Enfermería basada en evidencias, Advocacia em saúde, Enfermagem, Formação de conceito, Promoção da saúde, Enfermagem baseada em evidências

## Abstract

**Objective:**

To analyze the concept of Health Advocacy from the methodological framework of the Evolutionary Model.

**Method:**

The concept of interest was evaluated from the perspective of published studies identified in the databases: Web of Science, CINAHL, EMBASE, SCOPUS, MEDLINE and articles of interest. The attributes were determined from 19 scientific productions. Data were analyzed using thematic analysis, proposed by Bardin.

**Results:**

The following operational definition was obtained: Health Advocacy is an intentional action, implemented jointly and in favor of individuals and communities, especially for those who suffer from health inequalities, with the aim of preserving and improving health, well-being and empowerment for health promotion.

**Final considerations:**

Thus, a broader concept of Health Advocacy was abstracted, from the micro to the macro, which contemplates the development of the patient’s autonomy; includes individuals and groups in care plans and involves them in political activities as possibilities to provide assistance and correct health inequalities.

## RESUMO

**Objetivo:**

Analisar o conceito de Advocacia em Saúde a partir do referencial metodológico do Modelo Evolucionista.

**Método:**

O conceito de interesse foi avaliado na perspectiva dos estudos publicados e identificados nos bancos de dados: *Web of Science*, CINAHL, EMBASE, SCOPUS, MEDLINE e artigos de interesse. Os atributos foram determinados a partir de 19 produções científicas. Os dados foram analisados utilizando-se a análise temática, proposta por Bardin.

**Resultados:**

Obteve-se como definição operacional: a Advocacia em Saúde, configurada como ação de caráter intencional, implementada em conjunto e em prol de indivíduos e coletividades, especialmente para os que sofrem com iniquidades em saúde, com o objetivo de preservar e melhorar a saúde, o bem-estar e o empoderamento para a promoção da saúde.

**Considerações finais:**

Assim, foi abstraído um conceito de Advocacia em Saúde mais abrangente, do micro ao macro, em que contempla o desenvolvimento da autonomia do paciente; inclui indivíduos e grupos nos planos de cuidados e envolve-os em atividades políticas como possibilidades de fornecer assistência e corrigir desigualdades em saúde.

## RESUMEN

**Objetivo:**

Analizar el concepto de Abogacía de Salud a partir del referencial metodológico del Modelo Evolucionista.

**Método:**

El concepto de interés fue evaluado bajo la perspectiva de estudios publicados e identificados en los bancos de datos: *Web of Science*, CINAHL, EMBASE, SCOPUS, MEDLINE y artículos de interés. Los atributos se establecieron a partir de 19 producciones científicas. Los datos se analizaron con el análisis temático, propuesto por Bardin.

**Resultados:**

Se obtuvo una definición operativa: La Abogacía de la Salud es una acción intencional, implementada conjuntamente y a favor de individuos y colectividades, especialmente de aquellos que sufren inequidades en la salud, con el objetivo de preservar y mejorar la salud, el bienestar y el empoderamiento para la promoción de la salud.

**Consideraciones finales:**

Se abstrajo un concepto más amplio de Abogacía de la Salud, de lo micro a lo macro, en el que se contempla el desarrollo de la autonomía del paciente; incluye a individuos y grupos en los planes de atención y los involucra en las actividades políticas como una posibilidad de brindarles atención y corregir las desigualdades en salud.

## INTRODUCTION

Knowledge in nursing has its own foundations that differentiate it from other areas. Among these assumptions, one can mention the guidance for health promotion. This, as a practice, involves empowerment to increase control over the factors that influence and impact health^(1)^.

In this perspective, the Galway Consensus, agreed in June 2008, subsidizes the implementation of health promotion actions through the standardization of fundamental competences to promote health, divided into nine domains: Favoring change, Health Advocacy, Partnership, Communication, Leadership, Diagnosis, Planning, Implementation and Evaluation and research. The document also emphasizes that the values and principles of health promotion are based on health determinants, equity, social justice and respect for diversity^(2)^.

Among the nine domains of competences, there is the concept of Health Advocacy, which is defined in the document Core Competencies in Health Promotion (CompHp), endorsed by the European Union, as: Claiming with and in favor of individuals, communities and organizations to improve health, well-being and capacity building for action in health promotion^(1)^.

It is understood that concepts are built throughout history, bringing significant contributions to the construction of knowledge. They are formed by identifying features common to a class of objects or phenomena. They are dynamic and strongly influenced by public socialization and interaction, and their development varies according to the social context^(3)^.

The concept of Health Advocacy, although defined in the literature, has gaps in terms of establishing well-defined limits and differentiating it from other similar concepts, such as Patient Advocacy, Environmental Advocacy, Self-advocacy and Nursing Advocacy^(4,5)^. These concepts can be applied to advocacy exercised by a professional and directed at different individuals or groups and understood at macro and micro levels. Thus, the authors did not identify a study that would clarify this distinction, leading the concepts to be often used interchangeably.

Additionally, there is a need to expand the investigation of the concept of Health Advocacy specifically in the field of Nursing (the CompHP definition has a multidisciplinary character), since the Health Advocacy exercised expressly by nurses has particular characteristics. However, exploring this concept can make the discussion about its applicability in nursing practice scenarios visible, expanding the understanding of the term and highlighting its relevance^(6,7)^.

The study aims to analyze the concept of Health Advocacy based on the methodological framework of the Evolutionary Model in order to make it possible to clarify the term and its distinction from similar ones, establishing a theoretical basis to guide practice and future studies^(3)^.

## METHOD

### Study Design

Concept analysis study, built from the methodological framework of the Evolutionary Model. According to the model, concepts are ideas, being better defined based on their use and the description of their set of attributes. It is a continuous process, subject to change, depending on the context, time and situation. The analysis of a concept must follow an inductive method, in which a consensus on the term is identified, its historical aspects are evaluated and elements of agreement and disagreement are determined in different areas^(3)^.

The concept analysis comprises six activities: 1. Identification of the concept of interest and associated expressions, in order to select the most appropriate terminology; 2. Choice of scenario and sample. In a literature-based analysis, setting refers to the period and areas or type of literature to be included; the sample is best available described in the relevant or pertinent literature; 3. Data collection and management to: highlight the attributes of the concept; and its contextual bases, antecedents, consequences and sociocultural and temporal variations, in order to understand the situations in which the concept can be used; 4. Data analysis, in general, through thematic analysis; 5. Identification of an example, when applicable, in order to clarify the application of the concept of interest; 6. Interpretation of results, with determination of implications, hypotheses and contributions to the future or continued development of the concept^(3)^.

### Setting

In this study, the concept of interest in Health Advocacy was defined from the perspective of published and indexed studies in scientific databases: Web of Science, CINAHL, EMBASE, SCOPUS, MEDLINE/PUBMED and references to articles related to the concept. These databases were chosen due to the availability of productions with complex and detailed discussions on the topic. The CINAHL base was specifically chosen for its focus on Nursing and the others, given the efficiency of its coverage^(8)^.

### Selection Criteria

The following inclusion criteria were used: original studies, reflective or reviews studies related to the theme of Health Advocacy; published in Portuguese, English or Spanish. Opinion articles, letters to the editor, previous notes were excluded; duplicate studies; research protocols; studies that did not describe the concept of Health Advocacy; and studies unavailable digitally or physically in full. The term Health Advocacy was used to search, and 4,001 results were identified. Excluding duplicates, 1,561 studies remained, which were read title and abstract, using a total of 19 articles that met the aforementioned criteria in this study.

### Data Collection

For the follow-up of the study, a research protocol was adopted, which allowed its systematization, inductive reflective-critical reading and conceptual analysis. This protocol was chosen because it had already been used in another similar study, which also followed the activities described in the Evolutionary Model^(9)^. The research protocol consists of nine data collection indicators: reference; year of publication; country of origin; concept; attributes; background; consequences; substitute terms; related concepts; use of the concept and application over time.

The characterization of each of the elements proposed by Rodgers for concept analysis was used, as follows: Background - Aspects, situations and phenomena that contributed to the construction of the concept; Consequences - Refer to characteristics or phenomena generated after the construction of the concept; Substitute terms - Terms or expressions used that have the same meaning in articles; Related concepts - Concepts that contribute to the constitution of the evaluated concept; Attributes - Terms or expressions that characterize the concept^(3)^.

### Data Analysis

Data were analyzed using the thematic content analysis proposed by Bardin to carry out the analyzes and followed three phases: pre-analysis; exploration of the material and; treatment of results, inference and interpretation. The concepts extracted from each study were listed and the coding units were systematically identified in each text. Subsequently, these units were grouped into categories, called attributes. Finally, a single concept emerges from themes present in each category, listed considering the frequency and relevance of the inferred meaning of the coding units^(10)^. To clarify the application of the concept, the examples were demonstrated through the list of publications revealing the practical application of the concept, called empirical references^(4)^ and interpreted by contrasting the interdisciplinary findings.

Data were analyzed using content analysis, proposed by Bardin to carry out the analyzes and followed three phases: pre-analysis; exploration of the material and; treatment of results, inference and interpretation^(10)^. To clarify the application of the concept, the examples were demonstrated through the list of publications revealing the practical application of the concept, called empirical referents and illustrated concomitantly with the antecedents and consequences^(4)^. The results were displayed in tables and figures and interpreted by contrasting the interdisciplinary findings.

## RESULTS

**Chart 1:**
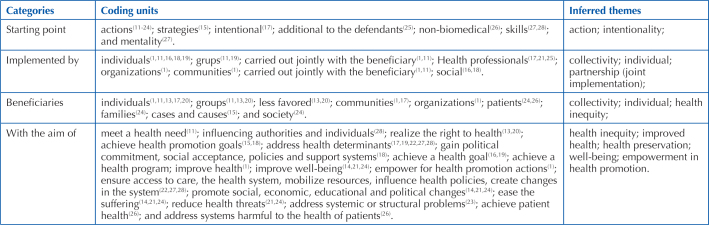
Attributes of Health Advocacy concept of - Fortaleza, CE, Brazil, 2022.

The attributes of the Health Advocacy concept were grouped into four parts: starting point of the concept, who implements it, who benefits from it and what it achieves (objective), as shown in Chart 1.

Most authors describe Health Advocacy as an action and some qualify or attribute some requirements to it, such as: intentionality; planning; not biomedical, necessarily; and that goes beyond the regular demands. In addition, another author emphasizes Health Advocacy as a job, since it is configured as any practice that involves intention, time and effort^(28)^.

There is reference to Health Advocacy as a mindset and skill^(27)^. However, since Health Advocacy is characterized as an action, it is understood that its existence is configured in a field of practice. It is understood that the stimulus for initiating the Health Advocacy action will depend on basic conditions, such as individual beliefs.

With regard to the characteristics of the action of Health Advocacy, it is a deliberate act. In this way, he must carry within himself the will to transform a reality, to intercede. Although it is understood that advocacy can manifest itself at macro and micro levels, there is a range of activities that should not be considered Health Advocacy^(13,28)^. An example of this occurs in the digital context, of social media, where a variety of topics are superficially discussed, without, however, presenting the potential to cause change.

The second group of attributes refers to the person responsible for implementing Health Advocacy. Different actors are reported in the literature, including individuals, groups, health professionals, organizations, communities and society. Therefore, it is understood that any individual or group can practice Health Advocacy. Authors emphasize that the action must be carried out jointly with the beneficiary^(1,11)^. Thus, two important components of the concept of Health Advocacy emerge, the participation and empowerment of those who have a place to speak on the subject.

Health advocacy implemented by nurses has particular characteristics, since it requires specific skills and actions from them, in addition to presenting facilities and barriers inherent in the area of nursing^(29)^. Corroborating this is the emphasis on the need to include training on Health Advocacy in the nursing curriculum^(28)^. Furthermore, the search using the terms “Health Advocacy” and “Nursing” showed that publications specifically using the terminology Health Advocacy in this area are rare.

Among the studies on Health Advocacy in the context of nursing, those focusing on actions at the micro level, directed at individual patients and with an emphasis on barriers related to interprofessional relationships and institutional conflicts of interest, stand out^(13)^. However, this type of advocacy, while necessary, has little potential for long-term change^(28)^. It is noteworthy that there are more vocal types of Health Advocacy and others that are more silent, so that health professionals tend to exercise the type that best suits their practices and lives^(14)^.

An example of Health Advocacy at the macro level would be actions to increase funding sources or change protocols for accessing gender-affirmative treatments by transgender people; and for micro advocacy it would be to help an individual patient who does not meet all the requirements present in a given protocol to gain access to gender reassignment treatment^(28)^.

When nurses practice advocacy, their own power, professional status, and job satisfaction are enhanced^(5)^. They can be inspired to face contemporary and future challenges, reflecting on the work of nursing personalities, such as Lillian Wald, Dorothea Dix and Florence Nightingale. These historic leaders embraced and shaped the delivery of population-based health care and advocacy and policy making^(29)^.

This is followed by the division of attributes with the detailing of the beneficiaries of the Health Advocacy action. The terms individuals and collectivities, likewise, broadly cover all beneficiaries, even in cases where the action will be in favor of cases and causes. By way of clarification, cases such as advocacy exercised at micro levels are understood, in general, advocacy directed at a patient; causes, on the other hand, is configured in advocacy exercised at the macro level, with objectives that will benefit collectivities.

Attributes two and three may be responsible for the variability of concepts related to Health Advocacy. It was observed that, in the context of health, there is a tendency to name the type of advocacy based on who performs it or benefits from it. The use of the term patient advocacy is frequently observed, for example, when the action is directed at a sick person; or the term advocacy in nursing when the action is implemented by nurses. These different usages result in confusion in the understanding and application of the terminologies.

The concept of patient advocacy has been discussed previously^(4,5)^. However, it is commonly used interchangeably with Health Advocacy, even though Health Advocacy is not restricted to the benefit of patients. Therefore, the existence of a conceptual hierarchy is assumed.

Finally, the attributes related to the objectives to be achieved were grouped together. When it comes to the desired result, it is observed that some authors do not distinguish between recommendations for how to reach the intended objective itself. For example, authors state that health advocacy is an intentional action by health professionals to address health determinants that negatively impact individuals or the community^(17)^. Other authors describe health advocacy as a combination of individual and social actions aimed at obtaining political commitment, social acceptance, policies and support systems^(18)^. It is observed that in both examples they cite mechanisms and omit the actual purpose of the activity.

In any case, a variety of different objectives were identified, which included: (a) structural changes, such as obtaining political commitment, social acceptance, policies and support systems, influencing authorities and individuals, addressing systemic problems, promoting social changes, economic, educational, and political, address health determinants, mobilize resources, influence health policy, create system change, reduce health threats, and address unhealthy systems; (b) curative perspectives, aiming to alleviate suffering, achieve health, guarantee access to care and the health system, implement the right to health and meet a health need; and (c) preventive perspective and health promotion, to achieve health promotion objectives, with a view to developing health promotion actions; and improve health and well-being.

Knowing that health is socially determined, for example by economic conditions, housing, food; actions to result in real impact on health require structural changes. At the same time, considering the range of demands, it is important that the strategies consider the need for health equity^(28)^. In Brazil, equity is one of the principles of the health system and should be taken into account in the management of health actions and resources.

Health Advocacy is based on the values of health promotion. Thus, a broader concept of health is assumed, contemplating individual perspectives and empowerment to maintain health. For example, when there is a movement to implement culture, leisure and sports activities in the community for the elderly population, there is a demand for health, instead of exclusively curative.

Therefore, from the determination of the most relevant attributes, the operational definition was arrived at: Health Advocacy is configured as an intentional action, implemented jointly and in favor of individuals and collectivities, especially for those who suffer from inequities in health, with the aim of preserving or improving health, well-being and empowering for health promotion.

**Chart 2:**
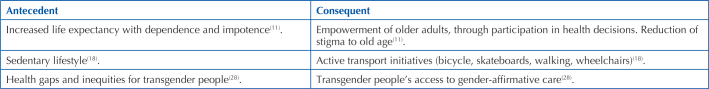
Empirical reference of the concept of Health Advocacy, example of antecedents and consequences - Fortaleza, CE, Brazil, 2022.

**Figure 1:**
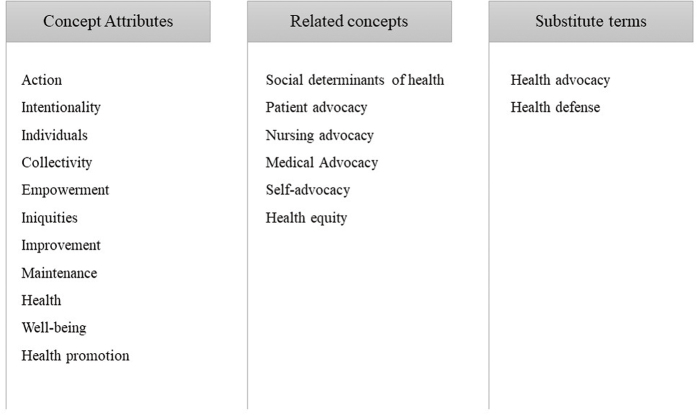
Concept attributes, related concepts, and substitute terms for Health Advocacy – Fortaleza, CE, Brazil, 2022.

Chart 2 presents empirical references to the concept of Health Advocacy, detailing the antecedents and consequences, and Figure 1 includes the main attributes, related concepts and substitute terms.

## DISCUSSION

Although the terminology Health Advocacy appears in studies published from 1974 to 1993, the publications referred to the term without proceeding with its previous definition or continuing with the concept of advocacy only. Some researchers have tried to construct a clear definition or framework for understanding Health Advocacy; however, these efforts met with limited success. As a result, there remained a divergence of understandings and wide variability in the enactment of Health Advocacy^(25,30)^.

The World Health Organization in the Glossary of Health Promotion defines Health Advocacy as the combination of individual and social actions to acquire political engagement, policy support, social acceptance and support of systems or programs to achieve goals^(16)^. It is observed that the concept emphasizes examples of mechanisms through which Health Advocacy manifests itself but fails to highlight elements of empowerment and detailing the characteristics of the objectives to be achieved.

In the Brazilian context, the Health Reform can be considered a Health Advocacy action, as it sought to reduce social inequalities and structural changes in the health care model. However, even so, the greatest challenge for the realization of the right to health in Brazil is to develop health democracy, related to the effective participation of society in strategic decision-making that result in the realization of the universal, equal and integral right to health^(20)^.

The document Core Competencies in Health Promotion (CompHp), endorsed by the European Union, published in 2012, defines Health Advocacy as: Advocating with and on behalf of individuals, communities and organizations to improve health and well-being and to develop capacity for health promotion actions^(1)^. However, a model is lacking to explain the term. Some important elements can be added to the concept, such as the deliberate character of the action, the emphasis on health inequities, the objectives, in addition to aspects related to the maintenance of well-being or health. Additionally, the term collectivities, or groups, better encompasses other aggregations of individuals, such as health professionals.

Finally, the virtual era adds a new scenario, or even a new element to the concept of Health Advocacy: the location. While, in general, Health Advocacy actions are taking place at the level of institutions, especially those of direct health care, in recent decades, with the use of the internet and social networks, the digital context has gained preponderance. Characteristics such as democratization and the expansion of the reach of information offer this technology enormous potential to generate changes.

## FINAL CONSIDERATIONS

The study brings a broader concept of Health Advocacy, from the micro to the macro, in which it contemplates the development of the patient’s autonomy; includes individuals and groups in care plans and involves them in political activities as possibilities to provide assistance and correct health inequalities. In this way, understanding the concept of Health Advocacy makes nurses more empowered in terms of practice, defending patients and families, as well as favoring health reforms.

It is expected to contribute to a more consistent use of the concept of Health Advocacy, for its use prior to its operationalization in practice, since health advocacy encompasses a wide range of activities such as: encouraging patients to engage in healthy behaviors (e.g., quitting smoking and losing weight), helping patients navigate health care (e.g., identifying services and investigations), and engaging in systems and policy-level activism (e.g., health care coverage for refugees and reducing pollution) to provide assistance and correct health inequalities.
