# ERRATUM: Analysis of the health advocacy concept from the perspective of the evolutionary method

**DOI:** 10.1590/1980-220X-REEUSP-2023-0170eren

**Published:** 2024-02-19

In the article “Analysis of the health advocacy concept from the perspective of the evolutionary method”, with DOI code number: https://doi.org/10.1590/1980-220X-REEUSP-2023-0170en, published at Revista da Escola de Enfermagem da USP [online], v. 57: e20230170:

On the page 1, where it was written:

**DESCRIPTORS**

Health Advocacy; Nursing; Concept formation; Health promotion; Concept formation; evidence-based nursing.

**Should read:**

**DESCRIPTORS**

Health Advocacy; Nursing; Concept formation; Health promotion; Evidence-based nursing.

On the page 6, where it was written:

**DESCRITORES**

Advocacia em saúde; Enfermagem; Formação de conceito; Promoção da saúde; Formação de conceito; Enfermagem baseada em evidências.

**DESCRIPTORES**

Defensa de la Salud; Enfermería; Formacíon de concepto; Promoción de la salud; Formación de concepto; Enfermería basada en evidencias.

**Should read:**

**DESCRITORES**

Advocacia em saúde; Enfermagem; Formação de conceito; Promoção da saúde; Enfermagem baseada em evidências.

**DESCRIPTORES**

Defensa de la Salud; Enfermería; Formacíon de concepto; Promoción de la salud; Enfermería basada en evidencias

